# Sequence-based detection and typing procedures for *Burkholderia mallei*: Assessment and prospects

**DOI:** 10.3389/fvets.2022.1056996

**Published:** 2022-11-14

**Authors:** Hanka Brangsch, Harisankar Singha, Karine Laroucau, Mandy Elschner

**Affiliations:** ^1^Institute of Bacterial Infections and Zoonoses, Friedrich-Loeffler-Institut – Federal Research Institute for Animal Health, Jena, Germany; ^2^National Research Centre on Equines (ICAR), Hisar, India; ^3^Bacterial Zoonosis Unit, Animal Health Laboratory, French Food Agency (Anses), Maisons-Alfort, France

**Keywords:** *Burkholderia mallei*, detection, PCR, MLVA, SNP, cgMLST, genome sequencing, genotyping

## Abstract

Although glanders has been eradicated in most of the developed world, the disease still persists in various countries such as Brazil, India, Pakistan, Bangladesh, Nepal, Iran, Bahrain, UAE and Turkey. It is one of the notifiable diseases listed by the World Organization for Animal Health. Occurrence of glanders imposes restriction on equestrian events and restricts equine movement, thus causing economic losses to equine industry. The genetic diversity and global distribution of the causing agent, *Burkholderia* (*B*.) *mallei*, have not been assessed in detail and are complicated by the high clonality of this organism. Among the identification and typing methods, PCR-based methods for distinguishing *B. mallei* from its close relative *B. pseudomallei* as well as genotyping using tandem repeat regions (MLVA) are established. The advent and continuous advancement of the sequencing techniques and the reconstruction of closed genomes enable the development of genome guided epidemiological tools. For achieving a higher genomic resolution, genotyping methods based on whole genome sequencing data can be employed, like genome-wide single nucleotide polymorphisms. One of the limitations in obtaining complete genomic sequences for further molecular characterization of *B. mallei* is its high GC content. In this review, we aim to provide an overview of the widely used detection and typing methods for *B. mallei* and illustrate gaps that still require development. The genomic features of *Burkholderia*, their high homology and clonality will be first described from a comparative genomics perspective. Then, the commonly used molecular detection (PCR systems) and typing systems (e.g., multilocus sequence typing, variable number of tandem repeat analysis) will be presented and put in perspective with recently developed genomic methods. Also, the increasing availability of *B. mallei* genomic sequences and evolution of the sequencing methods offers exciting prospects for further refinement of *B. mallei* typing, that could overcome the difficulties presently encountered with this particular bacterium.

## *B. mallei* genomic characteristics and relation to *B. pseudomallei*

The causative bacterial agent of glanders, *Burkholderia mallei*, is an obligate pathogen that can be transmitted between animals, but in rare cases also infects human, making glanders a zoonotic disease. However, the main hosts are equids, e.g., horses, donkeys and mules, wherefore glanders has an explicitly strong economic impact in rural areas of regions where *B. mallei* is endemic, e.g., Brazil, Tukey, Pakistan and India (1). Developed countries, where glanders has been eradicated by adopting strict control policies and regulations, are at risk of re-introduction of the disease due to import of infected animals (2, 3). Therefore, glanders incidences also affect international trade, equestrian events and economic losses to horse industry.

For the reliable diagnosis and molecular epidemiological investigation of glanders cases, knowledge about genomic features of this bacterium as well as the availability of reliable identification and typing methods is of utmost importance. One of the first challenges in the study of this bacterium was the differentiation of *B. mallei* from its close relatives *B. pseudomallei* and *B. thailandensis*. Based on genotyping data, it was hypothesized that *B. mallei* evolved from a single *B. pseudomallei* ancestor (4), which would explain the difficulty in differentiating these species because of their high genomic congruence. This assumption was further substantiated by comparative genomic analyses (5, 6). The split from *B. pseudomallei* was probably initiated by the introduction of the ancestral strain to an equine host, followed by adaption processes. This evolutionary process was characterized by a marked reduction of the genome size due to the loss of genes which were dispensable for survival in the host (4–6). Thereby, the ability of *B. mallei* to survive in versatile living environments decreased and restricted the bacterium to a limited host range as genomic islands coding corresponding functions in *B. pseudomallei*, e.g., the production of antibiotic compounds and metabolic pathways (5), were lost, deeming *B. mallei* an obligate pathogen with limited survival outside the host.

**Figure 1:**
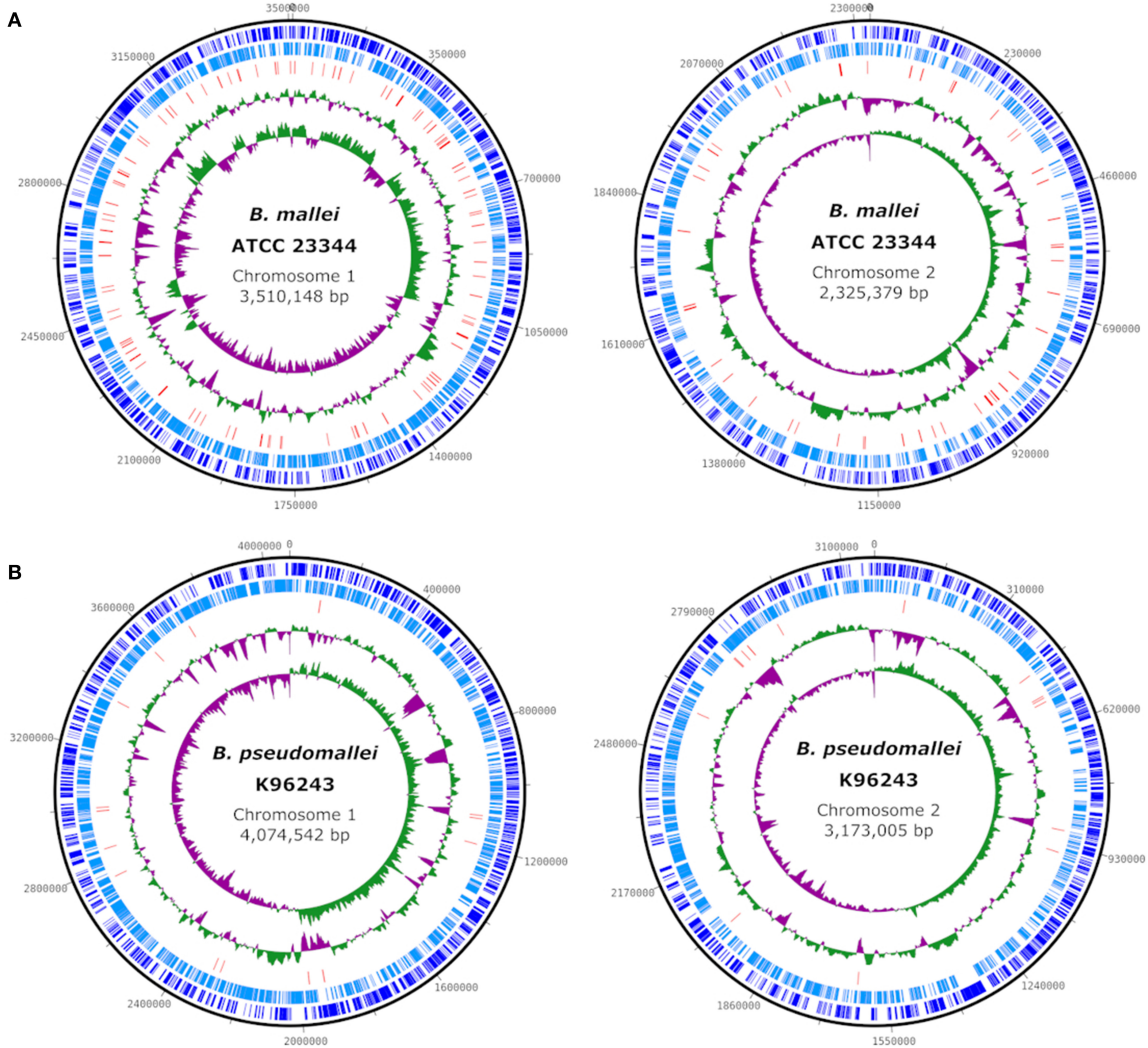
Comparison of the genome structure of the type strains *B. mallei* ATCC 23344 (CP000010, CP000011) **(A)** and *B. pseudomallei* K96243 (NC_006350, NC_006351) **(B)**. Circles (from outside to inside) show the location of coding sequences on forward (dark blue) and reverse (light blue) strands, predicted transposase-coding genes (red), GC content and GC skew. Plots were created using DNAPlotter v1.11 (7).

The genome of *B. mallei* has an average size of 5.7 Mb, which is about 1.5 Mb less than that of *B. pseudomallei* (Figure 1), and comprises two circular chromosomes. The main genetic divergence between both species results from this difference in genome size. Accordingly, the core genome of *B. mallei* is smaller and contains few species-specific genes, as 99% of the genome is identical to *B. pseudomallei*. However, the proportion of variable genes between *B. mallei* strains is higher than among *B. pseudomallei* strains, ranging between 33–41% and 11–22% of the genome content, respectively (5). Restriction of *B. mallei* to the host environment and the associated selection pressure also limits its genomic flux, which explains the small pan-genome (5).

The high degree of congruence between *B. mallei* and *B. pseudomallei* genomes had been demonstrated in DNA-DNA re-association experiments, even before both organisms had been assigned to the same genus (8). As DNA–DNA relatedness of the whole genome traditionally serves as basis for genus and species delineation (9), it was proposed that both species belong to the same genus, firstly as *Pseudomonas* species (8), and to the genus *Burkholderia* in 1992 (10). Even using more advanced DNA-DNA hybridization techniques, like microarrays, the differentiation between *B. pseudomallei* and *B. mallei* is difficult due to comparably high cross-hybridization signals between *B. pseudomallei* DNA probes and *B. mallei* DNA and vice versa (11). However, the differentiation of *B. mallei* and *B. pseudomallei* is reasonable for practical aspects as they differ in phenotypic traits, epidemiology and their zoonotic significance, i.e., zoonotic infections are exceedingly rare for *B. pseudomallei* (10, 12).

The high clonality reflects in a high average nucleotide identity (ANI), which can be determined *in silico* based on whole genome sequences. There are several approaches for delineating species boundaries based on this value and most of the times 96–97% of ANI are required for the definition of a species. For *B. mallei* the threshold lies well above 99%, being one of the highest ANI values (13–15). These high ANI values between *B. mallei* strains, but also to *B. pseudomallei*, poses a challenge to typing systems.

Genome sequencing revealed numerous insertion sequences (IS) of various IS families including transposase-coding genes in the genome of *B. mallei* (Figure 1), which are hypothesized to be a major driver of genome alteration compared to *B. pseudomallei* by promoting deletions, insertions and inversion mutations. The loss of several metabolic pathways that would enable the survival of the pathogen in the environment can be attributed to frameshift mutations caused by IS elements, as the genes are intact but inactive in *B. mallei*. IS-mediated recombination enabled continuous genome rearrangement and gene (cluster) deletion; however, this process is still continuing (5, 6). An important feature of the *B. mallei* genome is the exceptionally high density of simple sequence repeats, that might be important during the infection cycle (6), but which also complicates *in silico* analysis of the genome sequence, as will be described below.

## *B. mallei* molecular detection

**Figure 2:**
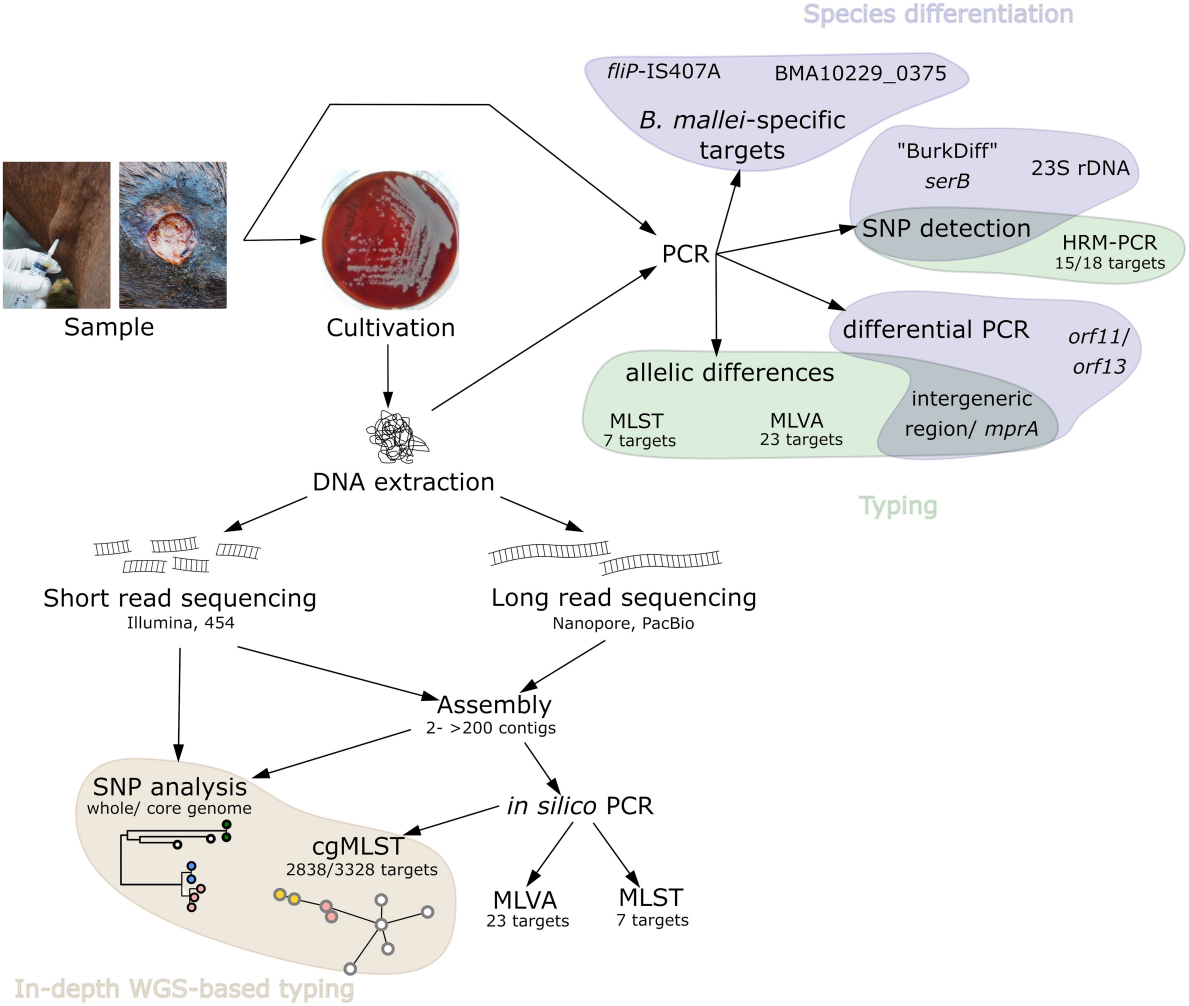
Schematic overview of the available, relevant PCR-based *B. mallei* detection and typing methods as well as methods employing genome sequencing.

For the diagnosis of *B. mallei*, reliable and quick differentiation of this organism from *B. pseudomallei* is an important step in case of suspected glanders infections. Due to the usually low number of bacteria in infected material (16), PCR appears to be the method of choice for an initial analysis of samples. Thus, for reliable differentiation of the two species, stable and phylogenetically informative markers are required which can be divided in different groups, depending of the type of approach used, e.g., SNP- or specific target gene-based (Figure 2). Differences in non-coding spacer regions have been identified (17), however, those regions can be assumed to be more prone to mutations, making them unsuitable for diagnostic purposes. Stable base substitutions that differentiate *B. mallei* from other *Burkholderia* sp. have been identified in the 23S ribosomal DNA and the phosphoserine phosphatase gene *serB*, based on which PCR assays have been developed, like the *serB*-based “BurkDiff” (18, 19) (Table 1). Further, the uneven distribution of genes encoding type III secretion systems (TTS) for the delivery of toxins to host cells can be employed in PCR assays. The absence of one out of three TTS-encoding genes, namely *orf11*, in *B. mallei* enabled the differentiation from *B. pseudomallei* (23) (Table 1). However, such assays have to be carefully validated. Even protein-coding regions, like the gene for flagellin C (*fliC*), were found to be unreliable due to point mutations in *B. pseudomallei* sequences which could lead to an incorrect classification of *B. pseudomallei* as *B. mallei* in the restriction digestion of the PCR product (20). Further, a multiplex PCR employing primers flanking a 10-bp repetitive element of varying size (400–700 bp) was evaluated for differentiation of *Burkholderia* species and strain differentiation of *B. mallei*, based on the presence of varying number of repeats found in different strains (24), which can also be used as a rough assessment of strain identity.

**Table 1:** PCR-based methods for differentiation of *Burkholderia* species.

**Target**	**Primers**	**Primers and probes**	Type*	**Remark**	**Source**
*fliC*	fliC-1 fliC-2	5'-GAT CGG CGG CAT GGT TCA GA-3' 5'-CCG AGC GTT GCC TGC AGA TTG TT-3'	C	*B. mallei* and *B. pseudomallei* will both give a PCR product; subsequent restriction analysis for further differentiation is possible	(20)
23S rDNA	CVMP 23-1 M 23-2	5'-AAA CCG ACA CAG GTG G-3' 5'- CAC CGA AAC TAG CA-3'	C	Detection of a single distinctive SNP in *B. mallei* 23S rDNA	(18)
*fliP*	Bma-flip-f Bma-flip-r Bma-probe	5'-CCC ATT GGC CCT ATC GAA G-3' 5'-GCC CGA CGA GCA CCT GAT T-3' 5'-FAM-CAG GTC AAC GAG CTT CAC GCG GAT C-3'-BHQ1	C, RT	Specific for B. mallei, targeting insertion sequence in *fliP* gene	(21, 22)
*serB*	for rev probe	5'-CGA GCG CAT CGT ACT CGT A-3' 5'-CAA GTC GTG GAT GCG CAT TA-3' 5'-FAM-CTG AAA CGC GCA GCG-3'-MGB	RT	Detection of a single SNP in *serB* distinctive for *B. mallei*	(19)
*orf11* *orf13*	PM122 orf11R orf11pro orf13f orf13R orf13pro	5'-ATC GCC AAA TGC CGG GTT TC-3' 5'-CAA ATG GCC ATC GTG ATG TTC-3' 5'-FAM-TCG GCG AAC GCG ATT TGA TCG TTC-3'-TAMRA 5‘-CAC CGG CAG TGA TGA GCC AC-3' 5'-ATG CTC CGG CCT GAC AAA CG-3‘ 5'-FAM-ACG CCC GTC GAA GCC CGA ATC-3'-TAMRA	RT	Differential identification: *B. mallei* is only positive for *orf13*, but not *orf11*, while *B. pseudomallei* is positive for both	(23)
Intergeneric *mprA*	SR1 SR5 14F5 14R5	5'-ACC GCG TAT GAA GGG ATG TC-3' 5'-ACG CGC ACG CAC CTG CTG AAC-3' 5'-ACC TGC TGC CGG GCT ACG ACT TCA-3' 5'-CAC CTT GCC GAC CCA CGA GAT GC-3'	C	Differential identification: *B. mallei* does not give an amplificated for 14F5/14R5; *B. mallei* strains can be differentiated based on amplicon size of SR1/SR5 products	(24)
BMA10229_0375	for rev probe	5'-CGT TCG AGC TCA GCA ACC TCG TTA-3' 5'-AAG CGG TGA TGG ACC GCT GTA T-3' 5'-Cy5 -CAG TAT CCA GGT TTC ACC GCG CTC GAC-3'-IAbRQ	QRT	Probe and primers used in combination with *orf11* and *fliC* primers/probes; *B. mallei* is positive in *fliC* and BMA10229_0375	(25)
BMA10229_0375 and downstream region	F3 B3 FIP BIP LF LB	5'-TGC ACC GGT ATC AGT CGG-3' 5'-GGA AGT CGG GAT TGT TCT CG-3' 5'-TTC ACT GCA AGC GTC AGG CGG CGT TTT ATC ACA AGC GGA C-3' 5'-ATC TGC CCG TCA TCG AAA TGC ACG ATG GAA TGG GTC TCA CG-3' 5'-GTT GCC GCG GCC GGG ATC-3‘ 5'-CTG GTG ATC ATG AAA ACG-3'	LAMP	Specific for *B. mallei* as the target is absent in *B. pseudomallei*	(26)

*C, conventional; RT, real-time; QRT, quadruplex real-time PCR; LAMP, real-time loop-mediated isothermal amplification.

**Figure 3:**
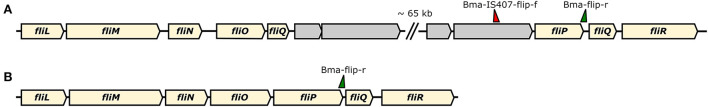
Schematic representation of the gene cassette for flagellum formation in *B. mallei* **(A)** and *B. pseudomallei* **(B)**. In *B. mallei* the *fliP* gene is disrupted by an IS407A element (gray arrows) that further promoted recombination, thus disrupting the cassette. Primer binding sites for differentiation of the two species according to the assay developed by Scholz et al. (21) are indicated by green/ red triangles.

The World Organization for Animal Health (WOAH) recommends the usage of two PCR-assays, a conventional PCR assay by Scholz et al. (21) and the real-time PCR assay by Tomaso et al. (22), which both take advantage of the numerous IS elements present in the *B. mallei* genome by targeting the flagellin P gene, *fliP*. This gene is disrupted in *B. mallei* by the IS407A element (6, 22), causing the immobility of *B. mallei*. This trait was exploited to design a PCR system overlapping *fliP* and IS407A for the species-specific identification of *B. mallei* (Figure 3) (21, 22). However, recently, this PCR has failed to diagnose glanders cases (27), possibly due to mutations in the primer/probe binding sites, loss of the IS407A insertion in the strain, or new recombination in this region of the genome. As the *B. mallei* genome continues to evolve through random IS-mediated recombination events (5, 28), vigilance is provided in the WOAH Manual of Diagnostic Tests and Vaccines for Terrestrial Animals (16), that such genetic evolutions within *B. mallei* could result in future variants that could escape detection by this PCR. Therefore, the development of robust, specific single-locus assays for diagnostics and the identification of different markers is recommended (27). For tracing glanders infections, even more refined methods have to be used in order to identify genotypes and differentiate strains of this highly clonal organism.

## *B. mallei* diversity

The molecular diversity of *B. mallei* strains has not been studied as extensively as that of other species of the genus *Burkholderia* (29, 30). Different molecular typing methods, originally developed for typing *B. pseudomallei*, have been applied to *B. mallei* strains because of their close genetic proximity. However, the differentiation of *B. mallei* strains, and thus the identification and linkage of infection sources, remains a challenge due to the very high homogeneity of the strains (14) composing this species. With the advancement of technologies, and in particular the complete sequencing of genomes, new perspectives are opening up.

### Pre-whole genome sequencing era

Various typing methods, including ribotyping, pulsed-field gel electrophoresis (PFGE), RAPD (random amplified polymorphic DNA), variable number of tandem repeat analysis (MLVA) and multi-locus sequence typing (MLST) were applied so far to *B. mallei* strains with varying degrees of discrimination.

The latter, which comprises the sequence analysis of seven highly conserved genes (MLST-7) (4, 5), revealed only one sequence type (ST40) for nearly all *B. mallei* strains, e.g., out of 120 investigated *B. mallei* strains, 118 strains were classified as ST40 while merely two strains, NCTC 10247 and NCTC 10260 isolated in Turkey, were identified as ST100 (31). Thus, this method is unable to depict the global diversity of *B. mallei* strains, merely allowing an identification of the organism at the species level. However, other typing methods have revealed greater diversity between *B. mallei* strains. Indeed, RAPD analysis, which is based on random amplification of genomic fragments by PCR and comparison of amplified bands on agarose gels (32, 33), proved to have a higher discriminatory power than MLST-7 as it enabled the identification of *B. mallei* clusters. However, clustering, and thus also the conclusions drawn on the basis of this method, heavily depends on the primers used for the initial amplification (33). Genotyping by different region (DFR) PCR targeting species-specific DNA sequences segregated 18 *B. mallei* strains into 11 types, which could be further clustered into two groups (34).

Methods based on enzymatic digestion of DNA fragments (ribotyping or PFGE), that were amplified in a targeted or non-targeted manner, have allowed the identification of distinct ribotypes or profiles within *B. mallei* strains. For example, ribotyping of 25 *B. mallei* isolates by gDNA digestion using the *Eco*RI and *Pst*I restriction enzymes followed by labeling with a probed oligonucleotide derived from the ribosomal RNA operon of *E. coli* revealed 17 distinct ribotypes (35), while typing of 21 *B. mallei* isolates by PFGE, after enzymatic digestion of the whole genomic DNA with *Spe*I, revealed 13 distinct types according to the resulting banding patterns (36). Again, the outcome of these typing methods heavily depends on the restriction enzymes used and the protocol was not widely evaluated for *B. mallei*. 16S rDNA sequence and ITS-based genotyping were able to discriminate *B. mallei* isolates circulating in India (37).

**Figure 4:**
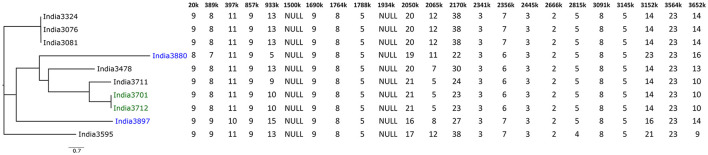
Example of MLVA-23 analysis of ten *B. mallei* strains from India (42). The allele number of each loci is given in the table. The Neighbor Joining tree was created using GrapeTree (44). Isolates colored in blue and green belong to HMR-PCR-based SNP group L2B2sB2_India-Group_1 and L2B2sB2_India-Group_2, respectively (45).

Although these methods are cost-effective and less labor intensive, their precision is limited in comparison to MLVA, a typing method initially developed from the first available *B. pseudomallei* genomes and based on the analysis of PCR amplified tandem repeats of 32 loci (MLVA-32). The initial MLVA typing study conducted on *B. mallei* included 21 strains from different geographical regions and identified 19 genotypes (38). However, a set of these initial loci were found to be dispensable for *B. mallei* typing due to their conservation within the species. Therefore, the MLVA scheme was reduced for *B. mallei* analysis to 23 loci (MLVA-23) corresponding to the variable markers for *B. mallei* (39) (Figure 4). This method has been applied for molecular investigation of glanders outbreaks in different countries (39–42). Recently, a restricted panel consisting of only six MLVA markers (loci 993, 3,145, 3,652, 20, 2,862, and 1,217) has been proposed (43). MLVA profiles can subsequently be used for comparative analysis using different algorithms, e.g., neighbor joining or minimum spanning analysis, that provide a graphical representation of the results and facilitate interpretation (Figure 4).

Among the different typing methods MLVA has been used most often to study the diversity of *B. mallei* strains and/or establish epidemiological links between glanders outbreaks. However, this method is time-consuming, costly and difficult to standardize.

### *B. mallei* whole genome sequencing

The advent of whole genome sequencing (WGS) techniques presented a new opportunity for gaining insight in genomes at an unprecedented resolution. The Illumina sequencing technology can be assumed to be currently the most frequently employed high-throughput genome sequencing method (46) as it allows sequencing at comparably low cost. The reads have lengths between 151 and 301 bp at a high accuracy (47), which is a major prerequisite for the correctness of the result generated by the sensitive genotyping methods in the subsequent analysis.

However, the short reads generated with this technology pose a challenge to the subsequent genome assembly process. The high number of repetitive sequences and the high GC content of *B. mallei* complicate the reconstruction of genomes: short reads cannot span long tandemly arrayed repeats that are longer than the read length, while genomic regions with a high GC content often exhibit low read coverage, primarily caused by PCR bias. These ambiguous genomic regions lead to gaps in the assemblies and thus to fragmented genomes comprising tens or hundreds of contigs (Table 2) (48–51).

**Table 2:** Overview of the recent status of *B. mallei* sequencing data deposited in the NCBI RefSeq public repository since 2004.

**Approach**	**Technology**	**Number of assemblies**	**Range of contig number**	**Mean contig number**
Individual	Illumina	27	2–627	252
	PacBio	15	2	2
	Roche 454	1	706	706
Hybrid	Roche 454 and Illumina	10	2–301	37
	PacBio and Illumina	9	2–3	2
	PacBio and Illumina and Roche 454	6	1–2	2
	Nanopore and Illumina	4	1–19	12
Missing data	Missing data	27	2–283	129

Entries for which no sequencing method was recorded, are specified as “missing data”.

The lack of contiguity in genome sequences based on Illumina reads can be overcome by sequencing technologies of the so-called third generation, that generate longer reads, like PacBio or nanopore sequencing technologies (47). Longer sequencing reads enable the construction of more contiguous and complete genomes, which is especially important for the detection of biosynthetic gene clusters and antibiotic resistance genes (49, 52). Nanopore sequencing proved to be a valuable tool for the quick detection of pathogens from clinical and environmental samples within 1 h of sequencing. It is highly productive and generates enough sequencing reads in 2–3 h for genome assembly (52, 53). This is a large improvement compared to Illumina sequencing runs, that can take up to 56 h before the sequence reads become available for downstream processing. However, one drawback of long-read technologies is the inherently higher error rate of the reads (49, 54). Especially genomic regions with consecutive repetitions of the same base or an array of bases pose a challenge for the sequencing process. In the case of Oxford Nanopore technology, where the actual sequencing signal is a change in electric current caused by the translocation of DNA through a pore, the exact length of base repeats can hardly be determined due to minor variation in the electrical signal. This frequently results in deletions in the final sequence and accounts for almost half of the sequencing errors (49, 54). Similar to PacBio technology, Nanopore sequencing helps generating less-fragmented genomes. However, as in case of Illumina sequencing, nanopore-based technologies are sensitive to high genomic GC content, which shows in a 2% higher error rate of such sequences as compared to low-GC reads (54).

In case of *B. mallei*, WGS not only helped elucidating the genome structure of the pathogen on a fine scale, but was also employed for unambiguously revealing the evolutionary relationship between *B. mallei* and *B. pseudomallei* at the genome level. The first *B. mallei* whole genome sequence was published in 2004 (6) and represented the type strain ATCC 23344. Based on this sequence, the description of genomic features like insertion sequences and gene clusters, core genome predictions, and the identification of virulence factors was carried out *in silico* (5, 6).

In an epidemiological context, the first study employing WGS for outbreak investigation was published in 2014 (40). Therein, isolates from the United Arab Emirates and Bahrain were compared attesting the close relationship of both populations.

In the NCBI RefSeq genome database, to date (September 2022), 99 *B. mallei* whole genome sequences are archived. Thirty-four of which are complete or on chromosome level, while 65 sequences comprise up to 706 scaffolds. These 99 sequences represent at most 74 unique strains. Due to missing comprehensive metadata, the exact number of strains remains elusive. Still, the impact of the sequencing technology on the completeness of the chromosomes is obvious (Table 2), although for 27 sequences the technology is not indicated (“missing data”). For individual sequencing technologies, Illumina-based assemblies remain highly fragmented: with the exception of one assembly, these genomes comprise 172–627 contigs (fragments). The Roche 454 technology, like Illumina, belongs to the second generation of sequencing platforms and generates short reads with an average length of 450 bp (55). Combining Illumina data with PacBio sequences markedly improves the contiguity of assemblies. However, a combination of likewise short reads derived from Illumina and Roche 454 technologies has limited success in closing genome gaps. At the time writing, merely four assemblies combining Nanopore and Illumina technologies are available.

### Whole genome sequencing-based typing

Despite the shortcomings of next generation sequencing technologies, they are valuable tools for outbreak investigations as they enable in-depth genotyping (Figure 2). In general, the methods employed can be roughly divided in gene-by-gene and SNP-based approaches (56). To date, whole genome sequencing has been mostly employed in *B. mallei* outbreak studies in addition to conventional methods such as MLVA (39, 40). Using a WGS approach, information about the number of repeats for each of the MLVA markers can be theoretically inferred from the genomic sequences. While this is commonly done for some pathogens, such as *Brucella* sp. (57), this approach has limitations for other bacteria such as *Bacillus anthracis* (58) and also *B. mallei* (59). Indeed, MLVA profiles obtained *in silico* from genomes of Pakistani *B. mallei* strains differed markedly from previously published profiles, disqualifying this *in silico* approach from sequencing data obtained by Illumina technology. As noted above, the assembly of the repeat regions, which are the targets of the MLVA, is highly prone to sequencing and/or assembly errors due to the short reads.

In order to overcome the inherent problems of the sequencing techniques and to achieve highly contiguous and accurate assemblies for reliable *in silico* gene-by-gene typing approaches, a combination of short- and long-read sequencing techniques should be considered. Thereby, errors in the long nanopore-generated sequences can be corrected with highly accurate Illumina reads, while the long reads can span genomic regions which the short reads alone cannot disambiguate (49, 60). In that way, an *in silico* MLVA approach may become feasible, as was shown for *Bacillus anthracis* (58).

Recently, a novel method ‘high-resolution melting' (HRM)-PCR which detects differences in the melting temperature of PCR amplified products due to single nucleotide polymorphism (SNP) in a target allele has gained attention for molecular epidemiological investigation of bacterial pathogens (61). It is developed based on whole genome sequences and combines the use of third generation DNA binding dyes, advanced real-time PCR platforms, and bioinformatics tools. The HRM-PCR is a single-step and closed-tube method thus offering simplicity, rapidity, versatility at low-cost.

Application of HRM-PCR for *B. mallei* typing was first introduced by Girault et al. (62) who described a set of 15 SNPs specific for each of the three phylogenetic clusters identified by Laroucau et al. (41). The HRM-PCR using this set of SNPs was validated with recently isolated *B. mallei* strains from India, Pakistan and Brazil (45, 62, 63) allowing further differentiation within the clusters. Recent *B. mallei* isolates from Dubai, UAE, Bahrain clustered in L2B1, strains from India and Pakistan clustered in L2B2sB2 whereas strains from Brazil clustered in L3B2. Thus, a connection between the detected HRM-PCR genotype and the geographic origin of the strains could be shown.

For a more in-depth phylogenetic analysis and in order to identify new informative SNPs for typing within the L2B2sB2 branch, a phylogenetic tree was reconstructed using WGS data of presently circulating strains in India and Pakistan. Four new SNP markers were selected, allowing to distinguish the Indian strains, and to differentiate two subgroups within this local group (45). This new set of markers was also applied for glanders positive clinical samples from Nepal (64). The results indicate that all samples clustered in the India_group 2 (large), which includes most of the Indian strains typed so far with this new set of markers, all originating from the northern Indian states of Uttar Pradesh and Haryana.

The markers determined to date have been based on the study of a small number of sequenced genomes, including few contemporary strains. This panel is enriched as new sequences become available and each time the relevance of the initial markers selected must be re-examined. A difference in the discriminatory power of HRM-PCR compared to MLVA can be seen in Figure 4, as the two strains India3880 and India3897, which were both assigned to L2B2sB2_India-Group_1 by HRM-PCR (45), differed in 10 MLVA loci (42), attesting the importance of MLVA for typing of outbreak strains from a restricted geographic area. HRM-PCR is a rapid typing method, targeting a limited number of markers that must be followed and eventually changed when new genomic sequences would no longer fit in the initial clusters determined. Therefore, the technology demands availability of genome sequencing data from different spatiotemporal regions.

A different WGS-based typing method that profits from contiguous and correct genome assembly is core genome multi locus sequence typing (cgMLST). A survey investigating the implementation of WGS and thereon-based techniques in European national public health reference laboratories found that cgMLST was the most frequently used typing approach (46). For this method, the classic MLST scheme is upscaled to thousands of loci for which the genome assembly is screened. Numbers are assigned to the associated allele sequences and allelic profiles are generated for each strain, which can be compared (56). Due to the standardized nomenclature, results are comparable between different laboratories. The allelic differences can be used to create a distance matrix which is further analyzed e.g., by neighbor joining algorithm (56, 65). Core genome MLST has been employed in tracing the infection sources of numerous pathogens, like *Listeria monocytogenes* and *Mycobacterium tuberculosis* (66, 67). Although cgMLST typing usually provides a slightly lower discriminatory power than SNPs (68), it can work well in concordance with SNP typing, depending on the relatedness of investigated strains, and help defining potential phylogenetic clusters (68, 69). Regarding *Burkholderia* sp., the first cgMLST schemes have been developed for *B. pseudomallei* and *B. stabilis* (70, 71). Recently, two cgMLST schemes were published for *B. mallei* (31, 59). Both used the type strain genome *B. mallei* ATCC 23344 as seed genome, but different sets of query genomes, resulting in the identification of different numbers of target genes: 3,328 and 2,838 core genome genes (66.2 and 56.5 % of the seed genome genes), respectively. Nevertheless, the discriminatory power of both schemes was demonstrated for the global *B. mallei* diversity as well as for local glanders outbreaks in Bahrain/ Dubai and Pakistan, respectively. By employing cgMLST analysis, geographical clusters of *B. mallei* could be identified. For example, three different lineages were detected in India, as well as different clusters in Bahrain and Dubai. On the other hand, the *B. mallei* population in Pakistan proved to be highly homogenic, which reflects the movement of equids between provinces (31, 59). Appelt et al. (31) additionally defined a three-allele threshold as the number of maximum allele differences between strains of the same outbreak.

A drawback of cgMLST analysis is the necessity of assembled genomes as basis for the analysis. Although Appelt et al. (31) state that assembly strategy would not change the cgMLST analysis result, it can be assumed that sequencing quality has a major impact on the assembly quality and thus also on a number of cgMLST targets. This was merely tested for two strains and has to be further assessed with a large number of sequencing data.

In order to prevent bias introduced by processing of the sequencing data, typing strategies that do not require genome assembly can be employed. Sequence-based SNP typing can be considered the gold standard for genotyping as it discriminates highly similar strains enabling detailed phylogenetic analysis (56). For *B. pseudomallei* it was shown that SNP typing provided a better phylogenetic resolution than MLST by revealing that identical MLST types from Cambodia and Australia were caused by homoplasy (72).

SNPs can be determined by either mapping reads to or by comparing assemblies with a reference genome and screening for nucleotide differences. When comparing multiple strains, a core genome SNP matrix is generated that includes all SNP positions that are present in all investigated genomes (56). Due to the high error rate in nanopore reads, the number of artificial SNPs detected in these sequences was high (49). Thus, the highly accurate Illumina-reads are more suitable for this analysis.

Few studies employed WGS-based SNP typing to *B. mallei* outbreak analysis. The agreement of SNP analysis with conventional VNTR analysis and HRM-PCR was proven for regional glanders outbreak events (40, 63), attesting the accordance of both methods on a small scale. Laroucau et al. (41) provided one of the first SNP-based analysis of the global *B. mallei* population. They identified three main lineages (L1-3) that exhibited a certain connection between genotype and geographic distribution: strains from Turkey were found to be highly similar, while strains from India were present in two lineages and altogether three branches. Likewise, two strains from Iran were assigned to different lineages. Due to the lack of available data, only 45 strains were included in this analysis, wherefore it can be supposed that a high proportion of the *B. mallei* genetic diversity remains to be detected. However, when comparing the clustering patterns generated by PCR-based MLVA and WGS-based SNP analysis, discrepancies become apparent as both lineages that were identified by SNP analysis intermingle in the MLVA results.

**Figure 5:**
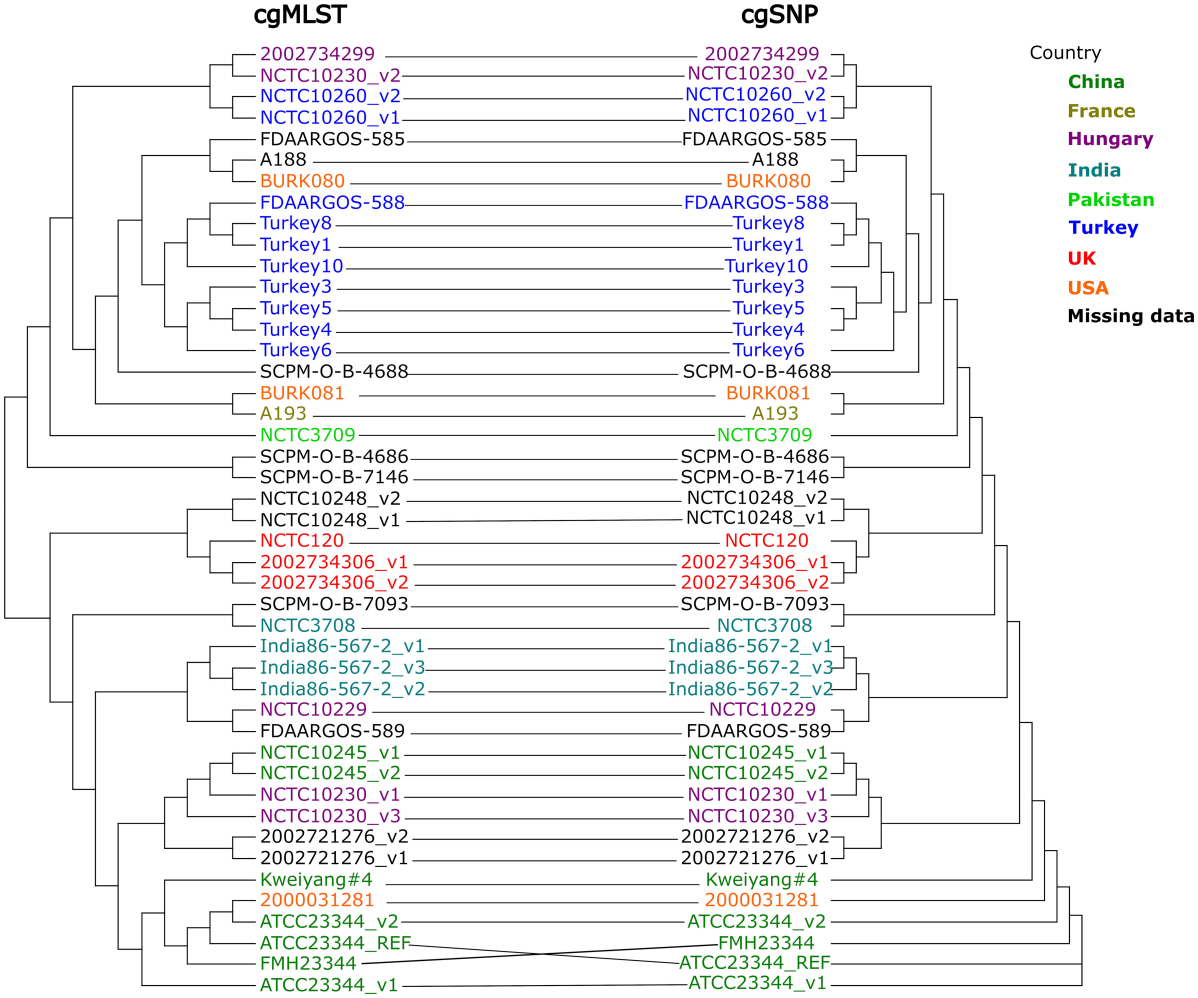
Comparison of clustering generated by cgMLST and cgSNP analysis (59) (modified). Colors indicate country of origin of the strain as registered with the corresponding BioSample entry.

The congruence of SNP and cgMLST analyses was demonstrated for a set of global *B. mallei* strains, which both methods clustered identically (59) (Figure 5). However, by employing SNP analysis, a more detailed strain differentiation is possible, as one cgMLST allele difference can reflect several base changes in the target.

SNP typing results depend immensely on various factors, like the choice of SNP calling strategy, reference strains and the strains included in the analysis, as they affect the number and location of detected SNPs. While one of the first studies merely included seven *B. mallei* strains and detected 515 SNPs, the inclusion of a larger number of strains and *B. pseudomallei* resulted in the detection of more than 9000 SNPs (5, 41).

## Perspectives for detection and typing

Taken together, several typing methods for *B. mallei* are at hand (Figure 2, Table 3), either based on PCR or WGS, providing different levels resolution. Whole genome sequencing has been implemented as a standard tool for outbreak investigations for many pathogens, nowadays. In contrast to PCR-based typing methods, no prior knowledge about the sample is required, as library preparation is mostly based on enzyme-based protocols, e.g., transposition, random PCR and ligation (47, 73). Using the DNA sequence reads generated by sequencing machines, bacterial genomes can be reconstructed and analyzed, e.g., for evolutionary and epidemiological studies. Thus, WGS enables in-depths comparative genomics where genome-wide variation can be detected at a high sensitivity and accuracy making it a valuable tool for molecular epidemiology. This high-resolution typing is required for unambiguous attribution of infection sources, infection chains and tracing of pathogens as sequences types of different strains can be assessed, provided that sufficient isolates and information is available. Thus, *in silico* analysis might completely substitute classical PCR-based approaches in the future (56), as MLVA and MLST-7 could be conducted based on the genome sequence (57, 74). Whether this is applicable to *B. mallei* has to be carefully assessed yet, as the construction of highly accurate genomes is a prerequisite for the application of these methods. It remains to be evaluated to what extent the high fragmentation of genome assemblies generated from Illumina reads can be solved by including data generated using nanopore sequencing, as a high contiguity of genomes is a prerequisite for these *in silico*analyses.

**Table 3:** Most important *B. mallei* typing techniques and relevant references of the methods or examples for application.

**Typing method**	**Technique**	**Targets**	**Remark**	**References**
MLST	PCR	Housekeeping genes	Low discriminatory power: *B. mallei* assigned to ST40 or ST100	(4)
MLVA	PCR	Tandem repeats	Useful for differentiation among strains of one outbreak, but targets are prone to homoplasy	(38, 39)
HRM-PCR	PCR	Canonical SNPs	Three large genotype groups and several subgroups can be differentiated	(45, 62, 63)
cgMLST	WGS	Nucleotide differences in core genome genes	In-depth differentiation based on assemblies	(31, 59)
cgSNP	WGS	Single nucleotide differences in core genome including intergeneric regions	In-depth differentiation based on sequencing reads or assemblies	(41, 59)

Although, compared to PCR-based methods, WGS analyses are more expensive and more complex in terms of reagents and equipment as well as bioinformatics analysis effort, the obtained sequences provide a higher information value, also for future questions, than PCR-based approaches. In addition, WGS methods will certainly become faster and less expensive in the future as these technologies will routinely be applied and bioinformatic analysis pipelines become more user-friendly.

MLVA will certainly remain an important tool for the differentiation of strains originating from a single outbreak event or a restricted geographic area. However, for investigating the global phylogeny of *B. mallei* these markers are not suited, as the comparably quickly mutating tandem repeats are prone to homoplasy (75). Thus, WGS-based methods should be preferred for phylogenetic analyses.

Different bioinformatic genotyping approaches have been developed for estimating the similarity between genomes. These can detect genome clusters and phylogenetic relationships based on which distance matrices and phylogenetic trees can be constructed allowing a quick and intuitive interpretation, even for non-bioinformaticians. A higher number of biomarkers can be detected at once and also new markers could be identified for refining the conventional typing methods (45, 62). Thus, WGS is an integral part for genotyping studies, especially for biothreat agents, and has been widely implemented in routine diagnostic analysis (46).

The on-going progress in sequencing technology development will surely provide the means for employing genome sequencing on a larger scale, even in low-budget settings. New algorithms for translating the electric current changes to base sequences (“basecalling”) that improve the sequence accuracy of nanopore reads are developed (54) and also the chemistry will change and improve in the future. However, this also demands for a constant adaption of analysis methods, protocols and bioinformatic software. This progress in sequencing technology will certainly impact comparative studies on *B. mallei* and there is a need for re-sequencing of historic and reference strains for obtaining high quality data. Improving the quality of sequencing data will enable the assembly of more contiguous genomes and prevent the loss of SNP positions, which can be overlooked if reads have a low quality due to the quality filters in SNP analysis.

In other pathogens, genome data is employed for the development of more specific DNA-based diagnostics, e.g., the development of a robust, highly specific PCR assay for differentiation of *Shigella* sp. from the closely related *E. coli* (76). Regarding *B. mallei*, analysis of genome data already has contributed to the improvement of conventional typing methods by revealing new phylogenetically informative SNPs that were incorporated in HRM-PCR analysis (45). Especially for the correct detection and identification of *B. mallei*, which is genetically homogenous and has high congruence with *B. pseudomallei*, WGS approaches might offer the possibility to develop assays with high specificity. However, a large set of diverse high-quality assemblies is required for ensuring the reliability of newly identified genomic targets and the targets have to be thoroughly validated in order to prevent false negative or false positive results (77).

Being a biothreat agent of the category B (78) and Tier 1 select agent, *B. mallei* is of special public concern as it could potentially be used in terrorist attacks. Thus, the possibility of the occurrence of genetically modified strains has to be considered. WGS would enable the quick detection of genetic manipulation leading to a gain of function, e.g., artificially introduced genes that increase virulence or pathogenicity. Further, the natural gain of resistances by mutations in housekeeping genes and the spread of genes can be detected and monitored by WGS, either based on assemblies or even on sequence reads (74, 79, 80). However, the prerequisite is the availability of a reliable database for quickly screening the WGS data, which has not been established for *B. mallei*, yet. There is a lack of studies on the connection between genomic features and phenotypic traits, that has to be addressed in future studies. Apart from genotyping, sequencing may allow insight in gene regulation and the prediction of connections between processes, e.g., by transcriptome sequencing, like quorum sensing in *Burkolderia* sp. (81).

Likewise, the lack of publicly available *B. mallei* sequences prevents comprehensive investigation of the global *B. mallei* genotype distribution as well as tracing of infection sources. This is not caused by a lack of awareness for the disease. In fact, most countries have strict regulations and control measure for preventing glanders outbreaks and the disease is listed in the WOAH Terrestrial Animal Health Code, meaning member states have to report cases to the organization. Nevertheless, isolation of strains is often not done or not successful due to a low bacterial load in clinical specimens (16). *B. mallei* strains are not systematically sequenced and if they are, sequence data is not systematically deposited in public databases and thus unavailable for comprehensive analyses. Likewise, incorrect database entries regarding strain identity and metadata have to be corrected. Appelt et al. (31) identified five entries where strain designation did not match the genotyping analysis result, which included two presumed type strains ATCC 23344 and Budapest (= NCTC 10229) (Table 4). This also applies to the metadata for raw sequencing reads deposited in the public repositories, as seen in Figure 5: one out of three sequencing datasets of strain NCTC 10230 does not cluster with the others. Further, strains isolated in Hungary were scattered across the trees in associations with strains from Turkey, China and India in this analysis, which rather points at incorrect metadata than veritable genotype diversity in Hungary. Further, the quality of the raw sequences as well as assemblies and annotations can be expected to be lower for older database entries. As the performance of bioinformatic tools is improving and new algorithms are being developed, a re-assessment of these old raw data is advisable.

**Table 4:** Strains with incorrect metadata deposited in public repositories (31).

**Strain designation**	**Accession number**	**Stated origin**	**Closest match**
ATCC 23344	NZ_CP008704.1, NZ_CP008705.1	China	NCTC 3709 (Turkey)
2000031063	NZ_CP008732.1, NZ_CP008731.1	Hungary	China5 (China)
FDAARGOS_587	NZ_RKJX00000000.1	Hungary	China5 (China)
KC_1092	NZ_CP009942.1, NZ_CP009943.1	Iran	China5 (China)
Budapest	LUFQ00000000.1	Hungary	SAVP1 (India)

To avoid the problems connected to isolation of bacteria, obtaining sequencing data directly from the sample material by employing a metagenomic approach could offer new possibilities in the future. A higher number and diversity of available sequences will allow a more detailed genotyping, as the choice of reference genome is of utmost importance for detection of informative SNPs. It is advised that instead of using a general reference genome, more detailed analysis can be enabled by utilizing a closely related strain as reference (82). Thus, it can be expected that WGS-based investigations of glanders outbreaks will give more reliable and detailed information if a suitable, i.e., closely related, reference is at hand, which will contribute to the understanding of *B. mallei* global genotype distribution.

## Author contributions

HS conceived the project. HB and ME coordinated the project, wrote the draft version of the manuscript, and prepared the figures. KL and HS also contributed to the text and revised the draft version. All authors approved the submitted version.

## Conflict of interest

The authors declare that the research was conducted in the absence of any commercial or financial relationships that could be construed as a potential conflict of interest.

## Publisher's note

All claims expressed in this article are solely those of the authors and do not necessarily represent those of their affiliated organizations, or those of the publisher, the editors and the reviewers. Any product that may be evaluated in this article, or claim that may be made by its manufacturer, is not guaranteed or endorsed by the publisher.
